# Behavioural Difficulties are Linked to Traumatization in Young Children: A Cross-Sectional Study in a Clinical Sample

**DOI:** 10.1177/13591045251398101

**Published:** 2025-11-15

**Authors:** Paulina Zelviene, Odeta Gelezelyte, Ask Elklit, Sille Schandorph Løkkegaard, Evaldas Kazlauskas

**Affiliations:** 1 Center for Psychotraumatology, Institute of Psychology, 54694 Vilnius University, Vilnius, Lithuania; 2 Danish Center of Psychotraumatology, Department of Psychology, University of Southern Denmark, Odense, Denmark

**Keywords:** story stem test, traumatization, young children, behavioural difficulties, OCTS

## Abstract

**Background:**

Exposure to traumatic events can have posttraumatic effects in young children. It is challenging to identify posttraumatic symptoms. It is necessary to directly investigate the signs of traumatization in young children. The aim of the study was to investigate the links between children’s externalizing and internalizing symptoms reported by caregivers and indicators of traumatization assessed by using the young children as informants.

**Method:**

Study used data from 110 (59.1% girls) children aged 4 to 9 years. The children have been recruited across the social and mental health services that provide support for abused children in Lithuania. Externalizing and internalizing difficulties measured using caregiver reports with the Strengths and Difficulties Questionnaire (SDQ). Indicators of traumatization were measured with the Odense Child Trauma Screening (OCTS).

**Results:**

The higher levels of indicators of traumatization were significantly related to higher conduct and externalizing problems. The traumatization indicators related to child mental representation of adults were a significant predictor of children’s conduct problems.

**Conclusions:**

The indicators of traumatization could be linked to externalized mental health difficulties of a child after trauma. Also, the findings might indicate that for the parents and caregivers it might be challenging to recognize internalizing symptoms, associated to traumatization.

## Plain Language Summary

Traumatic events are extremely difficult and life-threatening experiences that, studies show, may occur at any stage of life and might bring various psychological difficulties. Early recognition and identification of posttraumatic stress might help to prevent mental health difficulties later in life. However, while grown-ups can communicate and reflect on their experiences and feelings, this might be challenging for young children. It is important for practitioners and caregivers to understand better how trauma exposure is related to psychological problems in young children and how these difficulties can be recognized. Thus, this study examined the associations between behavioral and emotional difficulties and indicators of traumatization in young children. A newly developed, play-based, story stem tool, the Odense Child Trauma Screening (OCTS), was used for the recognition of traumatization. It contains LEGO^®^ house and dolls and can be used for young children aged 4 to 8 years. The dyads of 110 children and their caregivers were recruited from institutions that provide social and mental health services for children in Lithuania. The information on demographics and child difficulties were reported by caregivers, and potential indicators of traumatization were evaluated during individual contact with a child using the OCTS. It was found that child conduct problems, reported by the caregivers, were related to medium- and high-risk indicators of traumatization. Also, expectations of adult behaviour and reactions in the OCTS narratives significantly predicted conduct problems. This study broadens the understanding of child difficulties that might be related to the experienced psychological trauma. The findings of this study will benefit the development of psychological support services within countries with similar cultural backgrounds as Lithuania.

**Highlights**

• The study investigated the links between young children’s internalized and externalized difficulties reported by the caregivers and the risk for traumatization identified by young children (aged 4-8 years) using the Odense Child Trauma Screening (OCTS) measure.
• The study found significant links between conduct problems and the risk of traumatization in young children exposed to abuse. The indicators of traumatization as measured in a story stem test, especially those related to child mental representations of adults, could be linked to externalized mental health difficulties of a child, identified by the caregivers.
• Considering the lack of age-appropriate screening tools for young children’s traumatization, where the child is the primary informant, this paper provides important knowledge on the recognition of traumatization in young children.
• Story stem assessment tools such as the OCTS can be used to tap into the inner world of traumatized children.

## Introduction

Large numbers of young children are exposed to potentially traumatic events (PTE) (Briggs-Gowan et al., 2010a, 2010b; Lewis et al., 2019; Scheeringa, 2011) from various settings, e.g., neglect, physical and sexual abuse in foster care (Vasileva & Petermann, 2018), natural disasters (Scheeringa & Zeanah, 2008), domestic abuse (Levendosky et al., 2013) wars (Hodes, 2023) and others. Recent findings estimate that the risk for posttraumatic stress disorder (PTSD) for children aged 6.5 years and younger is 21.5% and that interpersonal and repeated traumas were significant risk factors for PTSD (Woolgar et al., 2022). Another systematic review also found that nearly one in four children and adolescents exposed to traumatic events may develop PTSD (Tamir et al., 2025). Besides PTSD, young children are at greater risk for developing other trauma-related emotional and behavioural difficulties (Briggs et al., 2021; de Young et al., 2011b; Haahr-Pedersen et al., 2024) and the symptoms may be less trauma-specific than in older children (World Health Organization, 2024). Clinicians working with children need to be aware of these findings to be able to provide appropriate and developmentally sensitive trauma-informed support.

Current diagnostic classifications have broad and developing definitions for young children’s trauma-related symptoms. The International Classification of Diseases 11th Revision (ICD-11) (World Health Organization, 2024) and the Diagnostic and Statistical Manual of Mental Disorders, Fifth Edition, Text Revision (DSM-5-TR) (American Psychiatric Association, 2022) state that children of all ages can develop trauma-related disorders such as PTSD. ICD-11 also describes complex PTSD (CPTSD) and that clinical expression of core posttraumatic symptoms for young children is vague, less specific and not necessarily trauma-specific (World Health Organization, 2024). Both ICD-11 and DSM-5-TR describe a wide range of inhibited and disinhibited behavioural and emotional symptoms: trauma-related reenactments (e.g., repetitive play, drawings), frightening dreams, symptoms of acting out, increased irritability, changes in mood, disturbed development, and others (American Psychiatric Association, 2022; World Health Organization, 2024). Also, traumatized children can present with other symptoms and problems than PTSD, such as symptoms of anxiety, depression, conduct disorders, school problems, social problems, and comorbidity is high (Briggs et al., 2021; de Young et al., 2011a; Haahr-Pedersen et al., 2024; Løkkegaard et al., 2017b). To sum up, there is a need for empirical data about trauma-related symptoms of young children (de Young et al., 2011a). This necessity is hindered by methodological challenges. Studies have demonstrated discordance between child and adult reports of child symptomatology. It was found, that parents tend to underreport internalizing symptoms compared to their children (De Los Reyes et al., 2015; Nader, 2007). Therefore, the establishment of age-appropriate assessment tools for young children and investigation of the links between adult and child reports might be essential for understanding the full picture of trauma-related difficulties.

Associations between adult-reported child behavioural difficulties and child-reported indicators of traumatization have been identified in previous studies (Futh et al., 2008; Wan & Green, 2010). The current study investigated these links using the Strengths and Difficulties Questionnaire (SDQ) to obtain caregiver reports of children’s difficulties, and the Odense Child Trauma Screening (OCTS) for children’s self-reported traumatization. The OCTS is a play-based story-stem tool that conceptualizes traumatization as play-based presentation of psychological difficulties following trauma exposure (Alkærsig et al., 2024). The same approach was applied in a sample of forty-nine Danish children (aged 4.5–8.9 years) from trauma-exposed and community samples. Significant medium correlations were found between the SDQ conduct and peer problems, hyperactivity, and the total score of the OCTS (Løkkegaard et al., 2021). Also, in a sample of 169 children aged 4-8 years from the general population, using the same tools, significant medium links were found between external difficulties (conduct problems and hyperactivity) and signs of traumatization for girls, whereas for boys there were no significant associations (Alkærsig et al., 2024).

The idea for the study comes from the recognition of a significant gap of knowledge about young children’s PTSD and trauma-related emotional and behavioural difficulties as well as from the need to understand the links between adult-reported child difficulties and young children’s reports. The aim was to explore the associations between the caregiver-reported externalizing and internalizing difficulties of the child and the indicators of traumatization, assessed through a story-stem measure in which young children served as informants. We were able to explore these links by using the Strengths and Difficulties Questionnaire (SDQ) (Goodman, 1997) and the Odense Child Trauma Screening (OCTS) (Løkkegaard et al., 2021) in the risk subsample of children recruited through social and mental health service centers for abused children in Lithuania.

## Methods

### Procedure and Participants

This study is part of a larger research project that examined the validity of the Lithuanian version of the Odense Child Trauma Screening (OCTS) (Zelviene et al., 2025). The current cross-sectional exploratory study included data from the risk subsample of children recruited through social and mental health service centers for abused children in Lithuania (*N* = 110). Data collection took place in the six organizations that work for potential child victims of abuse and with families at risk. Overall, twelve clinical psychologists working in the organizations collected the data. The study was approved by the Committee on research ethics in psychology at the Vilnius university. First, the psychologists distributed the informed consent form and information about the study to the legal guardians or caregivers of the potential child participants. Only those caregivers who signed informed consent were asked to participate in the caregiver-report survey using the paper-pencil method. After completing the caregiver-report survey, the trained psychologist obtained the child’s informed consent – explaining the procedure in a developmentaly appropriate manner – and then individually administered the OCTS. Caregivers could request feedback and were directed to psychological services if needed within the institution. The participant inclusion criteria were: (1) the age range of 4–8 years; (2) at least four completed stories of the OCTS according to the coding manual (Løkkegaard et al., 2018).

The sample included in the study comprised 65 (59.1%) girls and 45 (40.9%) boys. The average age was 6.78 (*SD* = 1.44), ranging from 4 to 9 years. There were eight 9-year-old children in the sample. The recommended age for administering the OCTS is 4-8 years (Løkkegaard et al., 2017a). However, as the OCTS scores of those 9-year-old children were not considered outliers, their data was included in the analyses.

Almost one-third of the risk sample participants (*n* = 35; 31.8%) lived with one parent, while 32 (29.1%) with guardians, 29 (26.4%) with both parents, 7 (6.4%) in an institution, 6 (5.5%) with other relatives, and 1 (0.9%) alternated between living with their mother and father. The majority (*n* = 87; 79.1%) had siblings. Regarding caregivers’ education, both caregivers of 26 children (24.8%) held a higher education degree, while one caregiver had a higher education degree in 28 cases (26.7%). The remaining caregivers did not have a higher education degree (*n* = 64; 31.4%). More than one-third of the participants (*n* = 38; 35.5%) had both caregivers employed, while 56 (52.3%) had one employed caregiver; the rest were both unemployed (*n* = 13; 6.3%). Alcohol or drug misuse was reported among the caregivers of 28 children (25.9%). Additionally, seven caregivers (6.5%) had a history of mental health illness, and 10 (9.3%) had a history of imprisonment. Half of the risk sample children (*n* = 55; 50.5%) had received professional psychological services (Zelviene et al., 2025).

### Measures

Caregiver reports included sociodemographic questions on the child’s age, sex, number of siblings, living situation, and previously received professional psychological help. It also included information on caregivers’ educational level, employment, substance misuse, history of mental illness, and imprisonment.

#### The Strengths and Difficulties Questionnaire (SDQ)

(Goodman, 1997) caregivers’ version (for 4-17-years-old children) was used to screen for the child’s psychological strengths and difficulties. The SDQ comprises four subscales to measure a child’s difficulties: Conduct problems and Hyperactivity subscales for externalizing symptoms, and Emotional problems and Peer problems subscales for internalizing symptoms. Each subscale includes five items, and each item is evaluated on a three-point scale (0 = Not true; 2 = Certainly true), based on a child’s behaviour over the last six months. The total scores of each subscale may range from 0 to 10, with higher scores on each of the four difficulties subscales indicating higher corresponding difficulties. The internalizing and externalizing difficulties were calculated by summing the corresponding subscales, and total scores may range from 0 to 20, with higher scores indicating greater difficulties. The total score of the SDQ is the sum of all the scores of all four difficulties subscales, which may range from 0 to 40, with higher scores indicating more serious difficulties. The SDQ was previously adapted to the Lithuanian population and showed good psychometric properties (Gintiliene et al., 2004). The internal consistency of the total SDQ in the present study was good Cronbach’s α = .82; acceptable for Emotional problems (α = .73) subscale, questionable for Conduct problems (α = .68) and Peer relationship problems (α = .61) subscales, and poor for Hyperactivity (α = .59) subscale. The internal consistency was acceptable for externalizing difficulties (α = .76) and internalizing difficulties (α = .74) symptom subscales.

#### The Odense Child Trauma Screening (OCTS)

(Løkkegaard et al., 2021) is a story stem and play-based, structured method that is designed to screen for signs of traumatization in children aged 4 to 8 years old. The OCTS includes a LEGO^®^ house and dolls representing family figures, which are used to administer the structured play. The tool comprises five main story stems that were played out and enacted by the OCTS administrator: a warm-up baseline story and four conflict stories, which included some distress for the child figure. The story stems builds up until the most critical point (e.g., the Nightmare story begins with all the characters sleeping in the middle of the night, when a child representative figure at some point wakes up from a bad dream); then, the child who is assessed, is asked to continue the story by telling and showing what happens next. By using the element of distress and enactment and play, the story stem tool taps into the child’s mental representation of self and others. The tool has an additional Animal story stem with several groups of animal figures (e.g., pigs, lions, giraffes), which can be used if the play with the human family figures does not provide sufficient material for reliable coding (Løkkegaard et al., 2021). However, in this study, all participants provided sufficient material for all main stories, and therefore, the Animal story was not included in these analyses.

The OCTS is administered by a trained and supervised psychologist who follows the instructions and story stem manuscripts from the administration manual (Løkkegaard et al., 2017a). The entire story stem assessment is videotaped for the subsequent coding. The administration of the OCTS usually takes around 20–30 minutes, with an additional 5–10 minutes if the Animal story stem is administered as well. At least four stories should be administered for the valid evaluation and calculation of a total score of the OCTS.

Child behaviour and narrative in each played story are coded using the OCTS coding manual (Løkkegaard et al., 2018), which was developed based on previous studies that used story stem methods in children exposed to traumatic events (Løkkegaard et al., 2021). The OCTS coding manual clearly guides the specialist in assigning the scores for 27 items for each of the four conflict story stem narratives (Løkkegaard et al., 2018). The coding system is based on previous studies that suggest that certain child mental representations are primarily linked to traumatization, while others are also found in children displaying symptoms of mood or behavioural disorders (Eriksen & Elklit, 2013; Løkkegaard et al., 2017b; Løkkegaard et al., 2018, 2021). The OCTS coding system and its 27 items are divided into three levels that are used to assess indicators of traumatization: 4 low-risk items (the items that are not directly related to traumatization but are included to describe general child engagement and compliance with the screening situation), nine medium-risk items (the items that represent characteristics or themes that are associated with possible traumatization or another vulnerability, e.g., narrative expression and mental representations of adults as controlling in times of child distress), and 14 high-risk items (the items that represent the child narratives that are highly likely to indicate traumatization, e.g., expression of sexual material in child narrative). All items are divided into five categories as well: (1) Child engagement and narrative production, (2) Nature of the narrative, (3) Adult representations in the narrative, (4) Child representations in the narrative, and (5) Disorganized phenomena. The structure of low, medium, and high-risk items within the categories are presented in Table 1. Child behaviour and narrative are coded using all 27 items for each story separately (Løkkegaard et al., 2018).

**Table 1.** Items and Total Scores Structure of the OCTS and Results in the Study Sample (*N* = 110)

OCTS items and categories	Number of items per story-stem	Total scores		OCTS total scores in the sample (*N* = 110)			
		*Min*	*Max*	*Min*	*Max*	*M*	*SD*
Traumatization risk							
High-risk items	14	0	112	0	43	9.58	8.92
Medium-risk items	9	0	72	0	38	17.85	9.58
Low-risk items	4	0	32	0	16	1.85	3.35
Engagement and narrative production							
Total	4	0	32	0	14	1.35	2.89
Low-risk items	3	0	24	0	12	0.95	2.38
Medium-risk items	1	0	8	0	6	0.40	1.32
Nature of the narrative							
Total	2	0	16	0	16	3.22	3.89
Low-risk items	1	0	8	0	8	0.90	1.85
Medium-risk items	1	0	8	0	8	2.32	2.57
Adult representations							
Total	6	0	48	0	29	11.58	6.24
Medium-risk items	3	0	24	0	17	9.08	4.13
High-risk items	3	0	24	0	14	2.50	2.87
Child representations							
Total	9	0	72	0	29	9.58	7.03
Medium-risk items	4	0	32	0	21	6.05	4.54
High-risk items	5	0	40	0	12	3.53	3.06
Disorganized phenomena	6	0	48	0	26	3.55	4.86

*Note*. OCTS – Odense Child Trauma Screening. *N* – total sample size, *M* – mean, *SD* – standard deviation, *Min*. – minimal value, *Max*. – maximum value.

Child engagement and narrative production category items are rated with dichotomous scores (0 = phenomenon is not present; 2 = phenomenon present). This category clarifies whether the administration and child narrative production are adequate for a reliable rating of the rest of the items for each story stem narrative. The remaining items are rated on a three-point scale (0 = phenomenon is not present; 1 = phenomenon expressed less clearly; 2 = phenomenon definitely present). Originally, the OCTS scoring and traumatization risk estimation is based on the weighted scores (Løkkegaard et al., 2018). In the current study, we used the raw scores of the OCTS. The total scores of the items representing different levels of traumatization and different categories were calculated by adding up all the corresponding items. Higher scores for risk items indicate a higher probability of experiencing traumatization, and for all categories, indicate higher expressed behaviour or narrative aspects related to the topic of the category. The possible range of total scores of the levels of traumatization and different categories are presented in Table 1.

The OCTS administration procedure in this study occurred within the institutions where the child was, with individual and confidential contact between the child and the trained psychologist. The psychologist prepared the evaluation materials: a LEGO^®^ house, figures, and video camera. Then, the specialist invited the child to take part in the testing in the child’s age-appropriate language. With the child’s informed consent, the psychologist guided them into the room, introduced the OCTS tool and procedure, and explained that the play session would be recorded. The psychologist monitored the child’s engagement throughout the session and discontinued the OCTS interview if the child chose not to participate. The Lithuanian version of the OCTS showed initial good psychometric characteristics in the Lithuanian sample (Zelviene et al., 2025).

### Data Analyses

The statistical analyses were carried out by using IBM SPSS Statistics 29. Spearman correlations among study variables of interest were calculated. We also conducted multiple regression analyses. No violations for assumptions of multiple regression (regarding multicollinearity, outliers, normality, etc.) were detected. For the comparison of sex and age groups, we used the Mann-Whitney U test. There were only a few missing data points, all for the SDQ scale. Specifically, 2.1% of data were missing for this scale. Two cases with all missing data were removed from the analyses that included the respective scale. As for low rates of missing values, individual mean imputation demonstrates good results (Shrive et al., 2006), the remaining missing scores were replaced by the mean of the subscale that an item represents.

## Results

### Associations Between the OCTS and the SDQ

The observed ranges, means, and standard deviations of the OCTS scores in a full sample are presented in Table 1. We analyzed the associations between the sum of the OCTS items representing low-, medium-, and high-risk traumatization and the child’s difficulties, which were assessed by the caregivers on the SDQ (Table 2). For each of the 27 items of the OCTS, raw scores assigned for four main story stems were summed. Then, the total scores of raw low-, medium- and high-traumatization risk items were calculated. Data from 108 children were included in the correlational analyses, as two caregivers did not fill in the SDQ questionnaires (Table 2). We found a statistically significant positive correlation between the SDQ Conduct problems subscale scores and the total scores of the OCTS high-risk items (*r* = .23, *n* = 108, *p* < .05) and medium-risk items (*r* = .37, *n* = 108, *p* < .001). Also, statistically significant positive correlations were revealed between the OCTS medium-risk items’ total score and the SDQ Externalizing problems subscale scores (*r* = .29, *n* = 108, *p* < .01) and SDQ total score (*r* = .26, *n* = 108, *p* < .01). There were no significant relationships between the OCTS risk items’ total scores and the SDQ Hyperactivity, Emotional Symptoms, Peer problems, and Internalizing problems scores (Table 2).

**Table 2.** Associations Between the OCTS High-, Medium- and Low-Risk Traumatization Items and the SDQ Subscales (*N* = 108)

	OCTS risk items			SDQ subscales					
	High	Medium	Low	Hyperactivity	Emotional symptoms	Conduct problems	Peer problems	Internalizing problems	Externalizing problems
OCTS risk items									
Medium	.68*** [.56, .78]								
Low	.15 [−.04, .34]	.38*** [.17, .54]							
SDQ subscales									
Hyperactivity	.06 [−.14, .24]	.16 [−.01, .32]	−.03 [−.20, .16]						
Emotional symptoms	.05 [−.14, .22]	.11 [−.08, .30]	.01 [−.17, .20]	.37*** [.19, .53]					
Conduct problems	.23* [.04, .42]	.37*** [.21, .54]	.06 [−.12, .26]	.57*** [.42, .71]	.33*** [.16, .50]				
Peer problems	.05 [−.17, .25]	.15 [−.04, .34]	−.01 [−.18, .17]	.23* [.05, .41]	.37*** [.18, .54]	.45*** [.27, .59]			
Internalizing problems	.08 [−.13, .27]	.15 [−.04, .34]	.00 [−.18, .19]	.41*** [.24, .55]	.87*** [.81, .91]	.48*** [.31, .62]	.75*** [.63, .84]		
Externalizing problems	.15 [−.05, .35]	.29** [.12, .45]	.02 [−.16, .22]	.88*** [.80, .93]	.40*** [.22, .56]	.89*** [.84, .92]	.38*** [.21, .52]	.50*** [.35, .63]	
Total	.12 [−.09, .32]	.26** [.08, .43]	.02 [−.15, .21]	.75*** [.63, .84]	.72*** [.59, .81]	.80*** [.70, .86]	.65*** [.51, .75]	.85*** [.77, .90]	.87*** [.82, .91]

*Note*. OCTS – Odense Child Trauma Screening; SDQ – Strengths and Difficulties Questionnaire. *n* – sample size. The 95% confidence intervals presented in brackets were calculated using a bootstrap method (1000 samples).

*p < .05, **p < .01, ***p < .001.

We further analyzed the relations between the different OCTS categories, sums of medium-risk and high-risk items within the categories, and the SDQ subscales (Table 3). We noticed the highest correlations among the SDQ Conduct problems and the OCTS Nature of the narrative, Adult representations in the narrative, and Child representations in the narrative categories total scores, correlation coefficients varied from .19 to .34. Thus, we conducted multiple regression analysis with the SDQ Conduct problems as the dependent variable and the OCTS categories total scores (Nature of the narrative, Adult representations in the narrative, Child representations in the narrative) as predictors (*n* = 108, *R*^*2*^ = 0.13, *F* (3, 104) = 5.39, *p* = .002). Of all the variables, only Adult representations in the narrative significantly predicted (*β* = 0.32, *p* = .018) the SDQ Conduct problems score.

**Table 3.** Associations Between the Different OCTS Categories and the SDQ Subscales (*N* = 108)

	OCTS engagement and narrative production	OCTS nature of the narrative	OCTS adult representations			OCTS child representations			OCTS disorganized phenomena
			Total	Medium-risk items	High-risk items	Total	Medium-risk items	High-risk items	
OCTS nature of the narrative	.24* [.05, .41]								
OCTS adult representations									
Total	.28** [.08, .45]	.42*** [.26, .55]							
Medium-risk items	.30** [.11, .46]	.42*** [.26, .55]	.94*** [.88, .97]						
High-risk items	.22* [.04, .41]	.37*** [.17, .54]	.87*** [.81, .91]	.66*** [.53, .77]					
OCTS child representations									
Total	.35*** [.15, .51]	.48*** [.33, .61]	.70*** [.58, .79]	.64*** [.50, .75]	.65*** [.51, .76]				
Medium-risk items	.37*** [.18, .53]	.46*** [.30, .59]	.59*** [.45, .70]	.54*** [.39, .67]	.54*** [.38, .68]	.94*** [.91, .96]			
High-risk items	.27** [.07, .44]	.43*** [.26, .56]	.69*** [.55, .79]	.63*** [.49, .74]	.66*** [.52, .78]	.85*** [.77, .91]	.64*** [.49, .75]		
OCTS disorganized phenomena	−.01 [−.19, .19]	.23* [.05, .42]	.44*** [.29, .57]	.31*** [.14, .48]	.51*** [.37, .65]	.41*** [.26, .56]	.28** [.10, .46]	.50*** [.36, .64]	
SDQ subscales									
Hyperactivity	.00 [−.18, .16]	.07 [−.13, .29]	.10 [−.09, .29]	.12 [−.09, .31]	.05 [−.15, .24]	.17 [−.01, .33]	.20* [.05, .36]	.06 [−.13, .24]	.04 [−.14, .22]
Emotional symptoms	−.06 [−.23, .12]	.11 [−.08, .29]	.10 [−.10, .29]	.14 [−.05, .33]	−.01 [−.19, .19]	.09 [−.11, .28]	.08 [−.11, .27]	.08 [−.11, .27]	−.01 [−.19, .17]
Conduct problems	.06 [−.13, .24]	.25** [.06, .42]	.32*** [.14, .49]	.34*** [.15, .50]	.20* [.02, .39]	.25** [.05, .43]	.27** [.09, .44]	.19* [−.01, .36]	.16 [−.06, .35]
Peer problems	−.05 [−.22, .13]	.15 [−.03, .32]	.13 [−.06, .32]	.15 [−.05, .34]	.04 [−.15, .22]	.03 [−.18, .22]	.10 [−.09, .28]	−.01 [−.19, .18]	.05 [−.15, .26]
Internalizing problems	−.09 [−.27, .09]	.15 [−.03, .32]	.14 [−.06, .34]	.17 [−.02, .36]	.03 [−.16, .23]	.10 [−.11, .29]	.11 [−.07, .30]	.07 [−.12, .26]	.04 [−.15, .24]
Externalizing problems	.05 [−.13, .22]	.16 [−.03, .34]	.22* [.04, .39]	.24* [.03, .43]	.13 [−.06, .32]	.22* [.03, .39]	.25** [.07, .42]	.13 [−.06, .30]	.09 [−.11, .28]
Total	−.01 [−.19, .16]	.18* [.01, .35]	.21* [.02, .39]	.24* [.04, .43]	.08 [−.10, .28]	.18 [−.02, .37]	.22* [.03, .40]	.10 [−.10, .28]	.06 [−.14, .24]

*Note*. OCTS – Odense Child Trauma Screening, SDQ – Strengths and Difficulties Questionnaire. *N* – sample size. The 95% confidence intervals presented in brackets were calculated using a bootstrap method (1000 samples).

*p < .05, **p < .01, ***p < .001.

### Comparison Between the OCTS Items Across Sex and Age Groups

We also compared the total scores of the OCTS items representing different categories and levels of traumatization risk across sex (Table 4) and age groups (Table 5). The statistically significant higher scores were revealed in boys in comparison to girls on medium traumatization risk items total score (*M* (*SD*) *=* 20.09 (9.87) and 16.31 (9.13) respectively, *p* = .038, *Z* = −2.08); and on Nature of the narrative category total scores (*M* (*SD*) *=* 4.07 (4.11) and 2.63 (3.64), respectively, *p* = .019, *Z* = −2.34). There were no significant sex differences on other items or categories.

**Table 4.** Comparison Between the OCTS Items and Categories Across Sex Groups (*N* = 110)

OCTS items and categories	Girls (*n* = 65)		Boys (*n* = 45)		*Z*	*p*	*r*
	M	SD	M	SD			
Traumatization risk level items							
High	9.23	9.16	10.09	8.64	−0.70	.484	.07
Medium	16.31	9.13	20.09	9.87	−2.08	**.038**	.20
Low	1.72	3.22	2.04	3.54	−0.58	.561	.06
Engagement and narrative production	1.20	2.71	1.58	3.14	−0.65	.519	.06
Nature of the narrative	2.63	3.64	4.07	4.11	−2.34	**.019**	.22
Adult representations							
Total	10.71	5.70	12.84	6.81	−1.55	.122	.15
Medium-risk items	8.49	4.06	9.93	4.13	−1.77	.076	.17
High-risk items	2.22	2.53	2.91	3.27	−0.89	.374	.08
Child representations							
Total	8.91	6.82	10.56	7.30	−1.14	.255	.11
Medium-risk items	5.71	4.34	6.56	4.82	−0.80	.426	.08
High-risk items	3.20	3.06	4.00	3.02	−1.54	.123	.15
Disorganized phenomena	3.82	5.51	3.18	3.74	−0.19	.847	.02
Age	6.89	1.43	6.62	1.47	−1.02	.307	.10

*Note*. OCTS – Odense Child Trauma Screening. *N* – total sample size, *n* – subsample size, *M* – mean, *SD* – standard deviation, *Z* – test coefficient, *p* – significance level, *r* – effect size.

Statistically significant differences are presented in bold.

**Table 5.** Comparison Between the OCTS Items and Categories Across Age Groups (*N* = 110)

OCTS items and categories	4-6 years (*n* = 44)		7-9 years (*n* = 66)		*Z*	*p*	*r*
	*M*	*SD*	*M*	*SD*			
Traumatization risk level items							
High	11.30	9.17	8.44	8.63	−2.04	**.041**	.20
Medium	20.34	10.07	16.20	8.93	−2.26	**.024**	.22
Low	2.14	3.43	1.67	3.30	−1.47	.142	.14
Engagement and narrative production	1.39	2.93	1.33	2.88	−0.18	.854	.02
Nature of the narrative	4.52	4.32	2.35	3.33	−3.15	**.002**	.30
Adult representations							
Total	12.57	6.65	10.92	5.91	−1.32	.186	.13
Medium-risk items	9.50	4.38	8.80	3.97	−1.00	.317	.10
High-risk items	3.07	2.99	2.12	2.74	−1.76	.078	.17
Child representations							
Total	11.25	7.58	8.47	6.47	−1.94	.053	.18
Medium-risk items	7.07	4.83	5.38	4.24	−1.96	**.050**	.19
High-risk items	4.18	3.28	3.09	2.84	−1.72	.086	.16
Disorganized phenomena	4.05	4.33	3.23	5.19	−2.21	**.027**	.21

*Note*. OCTS – Odense Child Trauma Screening. *N* – total sample size, *n* – subsample size, *M* – mean, *SD* – standard deviation, *Z* – test coefficient, *p* – significance level, *r* – effect size.

Statistically significant differences are presented in bold.

Regarding the age groups, we found that younger children (4–6 years old) performed statistically significantly higher scores in comparison to the 7–9 years-old group on overall OCTS high-risk traumatization items, (*M* (*SD*) *=* 11.30 (9.17) and 8.44 (8.63) respectively), medium-risk traumatization items (*M* (*SD*) *=* 20.34 (10.07) and 16.20 (8.93) respectively), Nature of the narrative category total score (*M* (*SD*) *=* 4.52 (4.32) and 2.35 (3.33) respectively), medium-risk items within the Child representations in the narrative category (*M* (*SD*) *=* 7.07 (4.83) and 5.38 (4.24) respectively), and Disorganized phenomena total score (*M* (*SD*) *=* 4.05 (4.33) and 3.23 (5.19) respectively). However, medium effect sizes were indicated between sex (*r* = .22) and age groups (*r* = .30) only when comparing scores of the OCTS Nature of the narrative. The rest of the effect sizes were small.

## Discussion

There is a gap in knowledge about young children’s PTSD and other trauma- and abuse-related emotional and behavioural reactions (de Young et al., 2011b). The aim of the study was to investigate the associations between caregiver-reported difficulties about the child and the risk for traumatization as indicated in a story stem assessment with children from a risk sample as informants. The current study was part of a research project that explored the validity and reliability of the newly developed Danish Odense Child Trauma Screening (OCTS) (Løkkegaard et al., 2021) in Lithuania (Zelviene et al., 2025). Since the OCTS coding system is rather broad, the current exploration was focused on items representing three levels (e.g., low, medium, and high risk) of traumatization and on items representing five narrative categories of the OCTS: (1) Child engagement and narrative production, (2) Nature of the narrative, (3) Adult representations in the narrative, (4) Child representations in the narrative, and (5) Disorganized phenomena (Løkkegaard et al., 2018) and their associations with Internalizing and Externalizing problems reported by the Strengths and Difficulties Questionnaire (SDQ) (Goodman, 1997).

Significant positive associations were found between the SDQ Conduct problems and the total scores of the OCTS high- and medium-risk traumatization items. Also, associations were found between the total scores of the OCTS medium-risk items, the SDQ Externalizing problems, and the SDQ total score. No associations were found between the total scores of the OCTS risk items and the total scores of the SDQ Hyperactivity, Emotional symptoms, Peer problems, and Internalizing problems. Out of all the associations, the SDQ Conduct and Externalizing problems stood out. Similar findings were found previously. In a sample of forty-six children from risk and community samples, the total score of the OCTS was associated with the total scores of the SDQ, Conduct, Hyperactivity, and Peer problems (Løkkegaard et al., 2021). It seems that for the caregivers it might be difficult to detect emotional and internalizing difficulties of the child. In summary, the results indicated preliminary findings on associations between behavioral difficulties and indicators of traumatization.

While exploring the associations between the SDQ difficulties and the OCTS narrative categories, we noticed associations between the SDQ Conduct problems and the OCTS Nature of the narrative, as well as Adult and Child representations in the narrative categories’ total scores. Thus, we conducted multiple regression analysis with the SDQ Conduct problems as the dependent variable and the OCTS categories’ total scores as predictors. Of all the variables, only Adult representations in the narrative significantly predicted the SDQ Conduct problems score in our risk sample. Trauma cannot only have a direct impact on children’s emotional and behavioural difficulties but can lead to attachment disturbances with the primary caregiver (de Young et al., 2011b). Initial findings suggest that indicators of traumatization, especially those related to child mental representation of adult behaviour and reactions in times of child distress, could be linked to externalized mental health difficulties of children in a risk sample.

The higher scores of the OCTS were found in boys compared to girls on total scores of the medium traumatization risk items and on the Nature of the Narrative Category items. There were no significant sex differences on other items or categories. We also found that in our study sample, younger children (4–6 years old) performed significantly higher scores in comparison to the 7–9 years-old group on overall the OCTS high- and medium-risk traumatization items, Nature of the narrative category total score, medium-risk items within the Child representations in the narrative category, and Disorganized phenomena total score. However, medium effect sizes were indicated between sex and age groups only when comparing scores of the OCTS Nature of the narrative. The rest of the effect sizes were small. The findings on age differences and, to some degree, also sex differences in the OCTS scores were indicated previously, especially the higher scores for boys and younger age children (Alkærsig et al., 2024).

### Limitations and Future Directions

Our study has several limitations that should be addressed. Information on the diagnostic status of mental disorders of the children included in the study was not available. In particular, about the PTSD diagnosis. However, PTSD is not routinely diagnosed for young children due to the diagnostic difficulties. With the progress of our understanding of the symptoms of PTSD in young children, future studies could explore symptom profiles and manifestations of PTSD, and the usage of measures such as the OCTS could provide insights into child traumatization and child mental representation of self and others. Future studies would benefit from a longitudinal design in order to explore the trajectories of change of symptoms after exposure to abuse and trauma. Also, given the cross-sectional design of the current study, the findings reflect associations rather than causality, pointing to the value of longitudinal research design for future studies. Furthermore, caregiver reports might be limited, as they were based on quantitative measures; incorporating qualitative data in the future studies could provide a valuable addition to the field. Multiple information sources about the child could provide more reliable data for future studies as well.

We also identified limitations related to the recruitment and study procedures. Since data were collected from different institutions, we were unable to track how many caregivers were invited to participate in the study. Therefore, it was not possible to report the response rate. Also, the research team collaborated with specific centers, therefore the results might be limited for generalizability. The findings also could be tested in other trauma-exposed child populations or diverse settings where the recognition of traumatization is needed, such as low-income, refugee, war affected contexts or other institutions or organizations. Additionally, some data were missing from the caregiver self-reports. However, the percentage of missing data was low, enabling us to select the most appropriate statistical approach to address this limitation.

### Conclusions

The aim of the study was to investigate the associations between caregiver-reported difficulties about the child and the risk for traumatization as indicated in a story stem assessment with children from a risk sample as informants. The study found that the higher levels of indicators of traumatization were statistically significantly related to higher caregiver reported conduct and externalizing problems. Only the traumatization indicators in the OCTS related to Adult representations in the narratives were a significant predictor of children’s conduct problems reported by the caregivers. We conclude that the indicators of traumatization, especially those related to child’s mental representation of adults in situations related to child distress, could be linked to externalized mental health difficulties of children in a risk sample. Although the study is cross-sectional, it can still inform policy, developmental pathways, and intervention methods for clinicians working with young children at risk for traumatization. The initial data calls for further investigation of these connections since it can help address the mental health challenges in young children who have experienced trauma such as abuse.

## Data Availability

The datasets of the current study are not publicly available for ethical reasons and data protection.*

## Abbreviations

OCTS: Odense Child Trauma Screening
PTSD: Posttraumatic Stress Disorder
SDQ: Strengths and Difficulties Questionnaire.

## References

[bibr1-13591045251398101] Alkærsig M. Elklit A. Løkkegaard S. S. (2024). Preliminary Danish norms for the odense child trauma screening (OCTS). Journal of Child & Adolescent Trauma, 17(3), 805–829. 10.1007/s40653-024-00616-7 39309336 PMC11413271

[bibr2-13591045251398101] American Psychiatric Association . (2022). Diagnostic and statistical manual of mental disorders. (5th ed.).

[bibr3-13591045251398101] Briggs E. C. Nooner K. Amaya-Jackson L. M. (2021). Assessment of PTSD in children and adolescents. In Friedman M. J. Schnurr P. P. Keane T. M. (Eds.), Handbook of PTSD. Science and practice (pp. 299–313). Guilford Publications.

[bibr4-13591045251398101] Briggs-Gowan M. J. Carter A. S. Clark R. Augustyn M. McCarthy K. J. Ford J. D. (2010a). Exposure to potentially traumatic events in early childhood: Differential links to emergent psychopathology. The Journal of Child Psychology and Psychiatry and Allied Disciplines, 51(10), 1132–1140. 10.1111/j.1469-7610.2010.02256.x 20840502 PMC3106304

[bibr5-13591045251398101] Briggs-Gowan M. J. Ford J. D. Fraleigh L. McCarthy K. Carter A. S. (2010b). Prevalence of exposure to potentially traumatic events in a healthy birth cohort of very young children in the northeastern United States. Journal of Traumatic Stress, 23(6), 725–733. 10.1002/jts.20593 21171133 PMC5972451

[bibr6-13591045251398101] De Los Reyes A. Augenstein T. M. Wang M. Thomas S. A. Drabick D. A. G. Burgers D. E. Rabinowitz J. (2015). The validity of the multi-informant approach to assessing child and adolescent mental health. Psychological Bulletin, 141(4), 858–900. 10.1037/a0038498 25915035 PMC4486608

[bibr7-13591045251398101] de Young A. C. Kenardy J. A. Cobham V. E. (2011a). Diagnosis of posttraumatic stress disorder in preschool children. Journal of Clinical Child and Adolescent Psychology, 40(3), 375–384. 10.1080/15374416.2011.563474 21534049

[bibr8-13591045251398101] de Young A. C. Kenardy J. A. Cobham V. E. (2011b). Trauma in early childhood: A neglected population. Clinical Child and Family Psychology Review, 14(3), 231–250. 10.1007/s10567-011-0094-3 21455675

[bibr9-13591045251398101] Eriksen S. B. Elklit A. (2013). Metoder til undersøgelse af mindre børns mulige traumer ud fra story stem-traditionen [Methods for assessment of younger children’s possible traumatization based on the story stem tradition]. Danish Center of Psychotraumatology, Department of Psychology, University of Southern Denmark.

[bibr10-13591045251398101] Futh A. O’Connor T. G. Matias C. Green J. Scott S. (2008). Attachment narratives and behavioral and emotional symptoms in an ethnically diverse, at-risk sample. Journal of the American Academy of Child & Adolescent Psychiatry, 47(6), 709–718. 10.1097/CHI.0b013e31816bff65 18434917

[bibr11-13591045251398101] Gintiliene G. Girdzijauskiene S. Cerniauskaite D. Lesinskiene S. Povilaitis R. Puras D. (2004). A standardised Lithuanian version of strengths and difficulties questionnaire (SDQ) for school-aged children. Psichologija, 29, 88–105. 10.15388/Psichol.2004..4355

[bibr12-13591045251398101] Goodman R. (1997). The strengths and difficulties questionnaire: A research note. The Journal of Child Psychology and Psychiatry and Allied Disciplines, 38(5), 581–586. 10.1111/j.1469-7610.1997.tb01545.x 9255702

[bibr13-13591045251398101] Haahr-Pedersen I. Vallières F. Hansen M. Aldamman K. Schmidt-Rasmussen V. Bramsen R. H. Spitz P. Hyland P. (2024). Evidence of a traumatic stress dimension of psychopathology among at-risk children living in Denmark. Current Psychology, 43(4), 3405–3415. 10.1007/s12144-023-04381-y

[bibr14-13591045251398101] Hodes M. (2023). Thinking about young refugees’ mental health following the Russian invasion of Ukraine in 2022. Clinical Child Psychology and Psychiatry, 28(1), 3–14. 10.1177/13591045221125639 36071016

[bibr15-13591045251398101] Levendosky A. A. Bogat G. A. Martinez-Torteya C. (2013). PTSD symptoms in young children exposed to intimate partner violence. Violence Against Women, 19(2), 187–201. 10.1177/1077801213476458 23420836

[bibr16-13591045251398101] Lewis S. J. Arseneault L. Caspi A. Fisher H. L. Matthews T. Moffitt T. E. Odgers C. L. Stahl D. Teng J. Y. Danese A. (2019). The epidemiology of trauma and post-traumatic stress disorder in a representative cohort of young people in England and Wales. The Lancet Psychiatry, 6(3), 247–256. 10.1016/S2215-0366(19)30031-8 30798897 PMC6384243

[bibr17-13591045251398101] Løkkegaard S. S. Andersen M. E. Eriksen S. B. Elklit A. (2017a). Odense child trauma screening: Administration manual. English version. Danish National Center of Psychotraumatology, Department of Psychology, University of Southern Denmark.

[bibr18-13591045251398101] Løkkegaard S. S. Egebæk S. A. B. Elklit A. (2017b). Are trauma and post-traumatic stress disorder connected to psychiatric comorbidity in Danish pre-schoolers? Journal of Child & Adolescent Trauma, 10(4), 353–361. 10.1007/s40653-017-0146-z

[bibr19-13591045251398101] Løkkegaard S. S. Andersen M. E. Eriksen S. B. Elklit A. (2018). Odense child trauma screening: Coding manual. English version. Danish National Center for Psychotraumatology, Department of Psychology, University of Southern Denmark.

[bibr20-13591045251398101] Løkkegaard S. S. Elmose M. Elklit A. (2021). Development and initial validation of the odense child trauma screening: A story stem screening tool for preschool and young schoolchildren. Scandinavian Journal of Child and Adolescent Psychiatry and Psychology, 9(1), 113–126. 10.21307/sjcapp-2021-013 34195104 PMC8216242

[bibr21-13591045251398101] Nader K. (2007). Understanding and assessing trauma in children and adolescents. Routledge. 10.4324/9780203940808

[bibr22-13591045251398101] Scheeringa M. S. (2011). PTSD in children younger than the age of 13: Toward developmentally sensitive assessment and management. Journal of Child & Adolescent Trauma, 4(3), 181–197. 10.1080/19361521.2011.597079 PMC6379904 30792828

[bibr23-13591045251398101] Scheeringa M. S. Zeanah C. H. (2008). Reconsideration of harm’s way: Onsets and comorbidity patterns of disorders in preschool children and their caregivers following hurricane katrina. Journal of Clinical Child and Adolescent Psychology, 37(3), 508–518. 10.1080/15374410802148178 18645742 PMC6087428

[bibr24-13591045251398101] Shrive F. M. Stuart H. Quan H. Ghali W. A. (2006). Dealing with missing data in a multi-question depression scale: A comparison of imputation methods. BMC Medical Research Methodology, 6(1), 57. 10.1186/1471-2288-6-57 17166270 PMC1716168

[bibr25-13591045251398101] Tamir T. T. Tekeba B. Mekonen E. G. Gebrehana D. A. Zegeye A. F. (2025). Shadows of trauma: An umbrella review of the prevalence and risk factors of post-traumatic stress disorder in children and adolescents. Child and Adolescent Psychiatry and Mental Health, 19(1), 48. 10.1186/s13034-025-00879-4 40301950 PMC12042603

[bibr26-13591045251398101] Vasileva M. Petermann F. (2018). Attachment, development, and mental health in abused and neglected preschool children in foster care: A meta-analysis. Trauma, Violence, & Abuse, 19(4), 443–458. 10.1177/1524838016669503 27663993

[bibr27-13591045251398101] Wan M. W. Green J. (2010). Negative and atypical story content themes depicted by children with behaviour problems. The Journal of Child Psychology and Psychiatry and Allied Disciplines, 51(10), 1125–1131. 10.1111/j.1469-7610.2010.02239.x 20331493

[bibr28-13591045251398101] Woolgar F. Garfield H. Dalgleish T. Meiser-Stedman R. (2022). Systematic review and meta-analysis: Prevalence of posttraumatic stress disorder in trauma-exposed preschool-aged children, 61, (3). 366–377. 10.1016/j.jaac.2021.05.026 PMC8885427 34242737

[bibr29-13591045251398101] World Health Organization . (2024). ICD-11: International classification of diseases (11th revision).

[bibr30-13591045251398101] Zelviene P. Gelezelyte O. Kairyte A. Elklit A. Schandorph Løkkegaard S. Kazlauskas E. (2025). Identifying traumatization in young children through structured play: Validation of the odense child trauma screening (OCTS) in Lithuania. European Journal of Psychotraumatology, 16(1), 2474373. 10.1080/20008066.2025.2474373 40063061 PMC11894742

